# The 50 most cited studies on trochleoplasty

**DOI:** 10.1002/jeo2.70183

**Published:** 2025-02-24

**Authors:** Alexander Pfarrmaier, Romed P. Vieider, Rodrigo Sanchez, Lukas N. Muench, Lukas Willinger, Sebastian Siebenlist, Armin Runer

**Affiliations:** ^1^ Department of Sports Orthopaedics Technical University of Munich Munich Germany

**Keywords:** bibliographic analysis, patella, patellofemoral Instability, qualitative analysis, trochleaplasty, trochleoplasty

## Abstract

**Purpose:**

This study aimed to analyse the 50 most cited publications on trochleoplasty (TP), examine their bibliographic parameters and evaluate the correlations between citation count, methodological quality and other factors.

**Methods:**

In a comprehensive literature search on the Web of Science, the 50 most cited studies on TP were identified. These studies were then evaluated according to their bibliographic parameters, level of evidence (LOE), citation counts, the Modified Coleman Methodological Score (MCMS), the Methodological Index for Non‑Randomised Studies (MINORS) and the Radiologic Methodology and Quality Scale (MQCSRE).

**Results:**

Of the top 50 list, 15 articles (30%) were published in the journal ‘Knee Surgery Sports Traumatology Arthroscopy’ (KSSTA). A total of 39 studies were published by institutes from Europe (78%), with France and Switzerland being represented 10 times each. Of eight different study types, case series (*n* = 25, 50%) and systematic reviews (*n* = 16, 32%) were the most prevalent. LOE included Level III (*n* = 1, 2%), Level IV (*n* = 41, 82%) and Level V studies (*n* = 8, 16%) studies. The total citation count amounted to 2481 citations, ranging from 10 to 187 (mean 49.6 ± 41.5) and showed a mean citation density of 5.1 ± 2.6. Quality scores were 60.8 ± 9.8 for MCMS (*n* = 26), 11.1 ± 2.9 for MINORS (*n* = 26) and 22.5 ± 2.1 for MQCSRE (*n* = 25), respectively. High citation counts did not statistically correlate with higher study quality scores (*p* > 0.05).

**Conclusion:**

Overall, there is growing scientific interest in TP as a treatment option for patients suffering from patellofemoral instability despite the lack of articles with a high LOE and methodological quality. This review of the top 50 most cited studies provides orthopaedic surgeons with a resource to assess the most impactful academic contributions to TP.

**Level of Evidence:**

Level IV.

**Abbreviations**
JCI: Journal Citation Indicator
JIF: Journal Impact Factor
LOE: level of evidence
MCMS: Modified Coleman Methodology Score
MINORS: Methodological Index for Non‐Randomised Studies
MPFL: medial patellofemoral ligament
MQCSRE: Radiologic Methodology and Quality Scale
PFI: patellofemoral instability
RPD: recurrent patellar dislocation
SR: systematic review
TD: trochlear dysplasia
TP: trochleoplasty
WoS: Web of Science by Clarivate™

## INTRODUCTION

Patellofemoral instability (PFI) is a pathology of multifactorial origin that can be caused by congenital or post‐traumatic anatomical and neuromuscular alterations [18]. It accounts for 2‐3% of all knee injuries, with an estimated incidence of 23.2/100.000 persons/year. The latter is highly age‐dependent, being 147.7/100.00 persons/year at 14‐18 years in comparison to 3.1/100.000 persons/year at ≥46 years [8, 22, 25].

Predictive factors for chronic PFI are trochlear dysplasia (TD), patella alta, TT‐TG > 20 mm, external tibial torsion, soft tissue hyperlaxity, malalignment, femoral anteversion, history of subluxation and an early onset of first patellar dislocation. Of these risk factors, TD is one of the most influential, with a hazard ratio of 18.5–23.7 [8, 18, 21, 22].

Dejour et al. [7] highlighted the critical role of TD as a significant predisposing factor in the pathogenesis of PFI, demonstrating that 96% of patients presenting with patellofemoral dislocation exhibited this anatomical abnormality. The surgical treatment of severe TD is often approached by performing a trochleoplasty (TP) with the primary goal to adjust the shape and depth of the trochlea in order to prevent patellar instability [25]. While the number of publications pertaining to TP for the treatment of PFI is increasing, a review evaluating the most impactful studies is useful to serve as a reference tool on this specific topic.

The aim of the present study was (1) to identify the 50 most cited studies on the topic of TP along with their bibliographic parameters and (2) to determine whether different factors, such as level of evidence (LOE), methodological quality, country of origin, journal of publication and different surgical techniques correlated with the citation count and citation density in the top 50 most cited studies.

## MATERIALS AND METHODS

### Literature search

A search of the Thomson Reuters Web of Science (WoS) database was conducted on 2 August 2024 to extract all available literature using the keywords ‘trochleoplasty’ and ‘trochleaplasty’ in the search category ‘topic’. WoS is a Clarivate™ platform that provides access to over 225 million records (as of May 2024), spanning more than 34,000 journals, books, proceedings, patents and data sets, covering a period from 1800 to the present day [1]. The WoS search yielded 381 records, published between 1970 and 2024, whose titles and/or abstracts included the terms ‘trochleoplasty’ or ‘trochleaplasty’. These records were compiled into a data sheet containing their title, author names, year of publication, journal, abstract, language and total citations (across all databases), then sorted by descending citation count. A similar search was performed using Elsevier's Scopus database for the terms ‘trochleoplasty’ and ‘trochleaplasty’ but since the results almost perfectly matched those from WoS, the ranking was based solely on the citation counts extracted from the WoS database.

### Inclusion and exclusion criteria

Of the initial list of 381 extracted records, the 154 least cited studies were excluded to focus on the most relevant research for the top 50. The titles and abstracts of the remaining 175 studies were then systematically screened, starting with the most cited. Studies were included if they met the following criteria: (1) TP as the primary topic, (2) TP performed specifically for the treatment of PFI and (3) not an animal or veterinary study. This selection process ensured that only the most relevant and impactful studies were considered for the final ranking.

**Figure 1:**
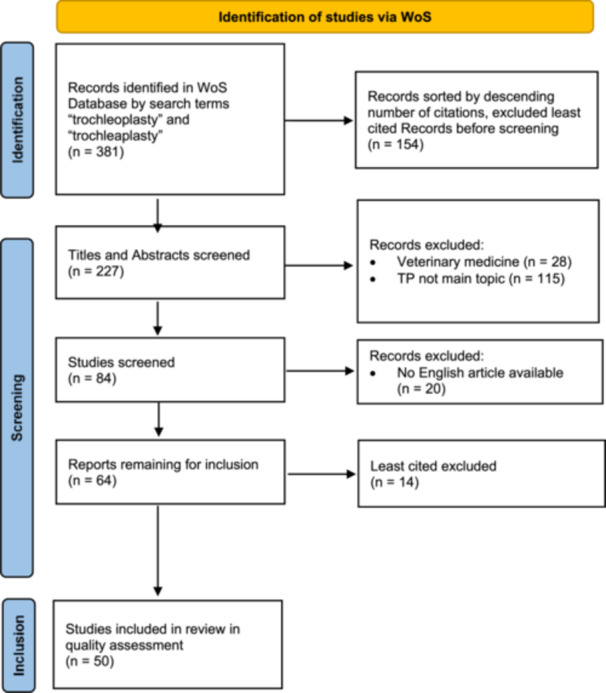
PRISMA flow diagram of the selection process of included records [16]. PRISMA, Preferred Reporting Items for Systematic Reviews and Meta‐Analyses; Wos, Web of Science.

This process yielded 84 articles, which were further screened. Subsequently, 20 records were excluded due to the absence of English versions. Of the remaining 64 articles, the 50 most cited were selected for final analysis (Figure 1).

### Data assessment

The included studies were evaluated based on the following criteria: first author, journal, 2023 Journal Impact Factor (JIF), Journal Citation Indicator (JCI), total citation count, study type, country of origin and year of publication. To ensure comparability between older studies, which typically accumulate more citations, and more recent articles, citation density (total citations divided by years since publication) was calculated.

Quality assessment included the LOE according to the Journal of Bone and Joint Surgery [24], the Modified Coleman Methodology Score (MCMS) [4], Methodological Index for Non‐Randomised Studies (MINORS) [20] and Radiologic Methodology and Quality Scale (MQCSRE) [2]. Interrater reliability was calculated for each score. The MCSM is a dependable tool to assess clinical studies, emphasising factors such as population size, follow‐up rates, diagnostic evaluations and rehabilitation protocols [4]. The MINORS score was created to quantify the methodological quality of observational studies, especially within the surgical fields [20]. As the main diagnostic tool for TD are radiological, such as common x‐rays, computed tomography (CT) or magnetic resonance imaging (MRI), the studies were also evaluated using the methodological quality scale designed for clinical studies involving radiologic examinations (MQCSRE) [2].

Systematic reviews (SRs), surgical techniques, cadaveric or biomechanical studies and histological examinations were not assessed, as the chosen scores were not developed for these types of studies.

### Statistical analysis

Descriptive statistics were calculated using SPSS (IBM, Version 27) and displayed by graphs and tables. The normal distribution of the variables was tested using the Kolmogorov–Smirnov test, which concluded that the different variables were not normally distributed. In cases where several groups were compared, the Kruskal–Wallis test was used, and in all other cases, the Mann–Whitney *U* test was used to compare single groups. A value of *p* < 0.05 was defined as statistically significant. The LOE and Quality scores (MCMS, MINORS and MQCSRE) were determined by independent raters and subsequently assessed using Pearson's correlation coefficient. Cohen's kappa (*κ*) was used to determine the agreement between the raters (*κ* < 0: no agreement; 0–0.20 slight, 0.21–0.40 fair, 0.41–0.60 moderate, 0.61–0.80 substantial and 0.81–1 almost perfect agreement [10]).

## RESULTS

### Descriptive statistics

**Figure 2:**
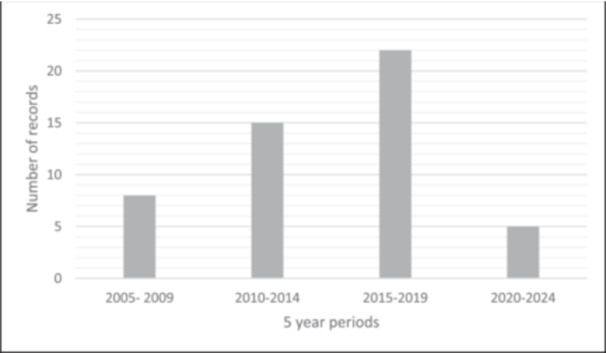
Absolute number of publications per 5‐year period.

The collective citation count of the top 50 most cited studies on TP resulted in 1870 citations, averaging 37.4 ± 34.2 citations per article (Table 1). The studies were published between 2005 and 2022, with the majority (*n* = 37; 74%) published between 2010 and 2019. For a more detailed evaluation and better illustration, 5‐year periods were analysed, showing almost half of the articles (*n* = 22) published between 2015 and 2019 (Figure 2).

**Table 1:** Comprehensive list of the 50 most cited studies on trochleoplasty (2 August 2024).

Rank (citations)	Authors	Year	Study design	LOE	MINORS	MCMS	MQCSRE
	*Title*						
1 (187)	**Dejour, D; Saggin, P** *The sulcus deepening trochleoplasty‐the Lyon's procedure*	2010	Surgical technique	5	N/A	N/A	N/A
2 (169)	**von Knoch, F; Böhn, T; Bürgi, ML; von Knoch, M; Bereiter, H** *Trochleaplasty for recurrent patellar dislocation in association with trochlear dysplasia: A 4‐to 14‐year follow‐up study*	2006	Case series	4	8	61	23
3 (134)	**Schöttle, PB; Fucentese, SF; Pfirrmann, C; Bereiter, H; Romero, J** *Trochleaplasty for patellar instability due to trochlear dysplasia: A minimum 2‐year clinical and radiological follow‐up of 19 knees*	2005	Case series	4	10	60	22
4 (127)	**Verdonk, R; Jansegers, E; Stuyts, B** *Trochleoplasty in dysplastic knee trochlea*	2005	Case series	4	8	67	N/A
5 (110)	**Donell, ST; Joseph, G; Hing, CB; Marshall, TJ** *Modified Dejour trochleoplasty for severe dysplasia: Operative technique and early clinical results*	2006	Case series	4	12	69	24
6 (101)	**Amis, AA; Oguz, C; Bull, AMJ; Senavongse, W; Dejour, D** *The effect of trochleoplasty on patellar stability and kinematics ‐ A biomechanical study in vitro*	2008	Biomechanical study	5	N/A	N/A	N/A
7 (100)	**Utting, MR; Mulford, JS; Eldridge, JDJ** *A prospective evaluation of trochleoplasty for the treatment of patellofemoral dislocation and instability*	2008	Case series	4	9	60	23
**8** (93)	**Ntagiopoulos, PG; Byn, P; Dejour, D** *Midterm Results of Comprehensive Surgical Reconstruction Including Sulcus‐Deepening Trochleoplasty in Recurrent Patellar Dislocations With High‐Grade Trochlear Dysplasia*	2013	Case series	4	10	49	20
9 (92)	**Nelitz, M; Dreyhaupt, J; Lippacher, S** *Combined Trochleoplasty and Medial Patellofemoral Ligament Reconstruction for Recurrent Patellar Dislocations in Severe Trochlear Dysplasia A Minimum 2‐Year Follow‐up Study*	2013	Case series	4	12	69	24
10 (75)	**Banke, IJ; Kohn, LM; Meidinger, G; Otto, A; Hensler, D; Beitzel, K; Imhoff, AB; Schottle, PB** *Combined trochleoplasty and MPFL reconstruction for treatment of chronic patellofemoral instability: a prospective minimum 2‐year follow‐up study*	2014	Case series	4	12	59	23
11 (73)	**Fucentese, SF; Zingg, PO; Schmitt, J; Pfirrmann, CWA; Meyer, DC; Koch, PP** *Classification of trochlear dysplasia as predictor of clinical outcome after trochleoplasty*	2011	Case series	4	11	81	24
12 (72)	**Balcarek, P; Rehn, S; Howells, NR; Eldridge, JD; Kita, K; Dejour, D; Nelitz, M; Banke, IJ; Lambrecht, D; Harden, M; Friede, T** *Results of medial patellofemoral ligament reconstruction compared with trochleoplasty plus individual extensor apparatus balancing in patellar instability caused by severe trochlear dysplasia: a systematic review and meta‐analysis*	2017	Systematic review and meta‐analysis	4	N/A	N/A	N/A
13 (61)	**Fucentese, SF; Schottle, PB; Pfirrmann, CWA; Romero, J** *CT changes after trochleoplasty for symptomatic trochlear dysplasia*	2007	Case series	4	8	39	15
14 (58)	**Rouanet, T; Gougeon, F; Fayard, JM; Remy, F; Migaud, H; Pasquier, G** *Sulcus deepening trochleoplasty for patellofemoral instability: A series of 34 cases after 15 years postoperative follow‐up*	2015	Case series	4	12	50	23
15 (58)	**Dejour, D; Byn, P; Ntagiopoulos, PG** *The Lyon's sulcus‐deepening trochleoplasty in previous unsuccessful patellofemoral surgery*	2013	Case series	4	10	52	21
16 (56)	**Blond, L; Haugegaard, M** *Combined arthroscopic deepening trochleoplasty and reconstruction of the medial patellofemoral ligament for patients with recurrent patella dislocation and trochlear dysplasia*	2014	Case series	4	13	64	22
17 (53)	**Testa, EA; Camathias, C; Amsler, F; Henle, P; Friederich, NF; Hirschmann, MT** *Surgical treatment of patellofemoral instability using trochleoplasty or MPFL reconstruction: a systematic review*	2017	Review	4	N/A	N/A	N/A
18 (48)	**Camathias, C; Studer, K; Kiapour, A; Rutz, E; Vavken, P** *Trochleoplasty as a Solitary Treatment for Recurrent Patellar Dislocation Results in Good Clinical Outcome in Adolescents*	2016	Case series	4	11	75	23
19 (46)	**Batailler, C; Neyret, P** *Trochlear dysplasia: imaging and treatment options*	2018	Review	4	N/A	N/A	N/A
20 (46)	**Song, GY; Hong, L; Zhang, H; Zhang, J; Li, X; Li, Y; Feng, H** *Trochleoplasty Versus Nontrochleoplasty Procedures in Treating Patellar Instability Caused by Severe Trochlear Dysplasia*	2014	Review	4	N/A	N/A	N/A
21 (46)	**Schottle, PB; Schell, H; Duda, G; Weiler, A** *Cartilage viability after trochleoplasty*	2007	Histological examination	5	N/A	N/A	N/A
22 (43)	**Ntagiopoulos, PG; Dejour, D** *Current concepts on trochleoplasty procedures for the surgical treatment of trochlear dysplasia*	2014	Review	4	N/A	N/A	N/A
23 (42)	**Blond, L; Schottle, PB** *The arthroscopic deepening trochleoplasty*	2010	Case series	4	9	69	24
24 (39)	**LaPrade, RF; Cram, TR; James, EW; Rasmussen, MT** *Trochlear Dysplasia and the Role of Trochleoplasty*	2014	Review	4	N/A	N/A	N/A
25 (38)	**Longo, UG; Vincenzo, C; Mannering, N; Ciuffreda, M; Salvatore, G; Berton, A; Denaro, V** *Trochleoplasty techniques provide good clinical results in patients with trochlear dysplasia*	2018	Review	4	N/A	N/A	N/A
26 (37)	**Hiemstra, LA; Peterson, D; Youssef, M; Soliman, J; Banfield, L; Ayeni, OR** *Trochleoplasty provides good clinical outcomes and an acceptable complication profile in both short and long‐term follow‐up*	2019	Review	4	N/A	N/A	N/A
27 (37)	**McNamara, I; Bua, N; Smith, TO; Ali, K; Donell, ST** *Deepening Trochleoplasty With a Thick Osteochondral Flap for Patellar Instability: Clinical and Functional Outcomes at a Mean 6‐Year Follow‐up*	2015	Case series	4	11	73	24
28 (36)	**Metcalfe, AJ; Clark, DA; Kemp, MA; Eldridge, JD** *Trochleoplasty with a flexible osteochondral flap RESULTS FROM AN 11‐YEAR SERIES OF 214 CASES*	2017	Case series	4	11	69	23
29 (35)	**Zaffagnini, S; Previtali, D; Tamborini, S; Pagliazzi, G; Filardo, G; Candrian, C** *Recurrent patellar dislocations: trochleoplasty improves the results of medial patellofemoral ligament surgery only in severe trochlear dysplasia*	2019	Systematic review and meta‐analysis	4	N/A	N/A	N/A
30 (34)	**Duncan, ST; Noehren, BS; Lattermann, C** *The Role of Trochleoplasty in Patellofemoral Instability*	2012	Review	4	N/A	N/A	N/A
31 (28)	**Nolan, JE; Schottel, PC; Endres, NK** *Trochleoplasty: Indications and Technique*	2018	Review	4	N/A	N/A	N/A
32 (28)	**van Sambeeck, JDP; van de Groes, SAW; Verdonschot, N; Hannink, G** *Trochleoplasty procedures show complication rates similar to other patellar‐stabilising procedures*	2018	Systematic review and meta‐analysis	4	N/A	N/A	N/A
33 (25)	**Ren, B; Zhang, X; Zhang, L; Zhang, MY; Liu, Y; Tian, B; Zhang, BH; Zheng, J** *Isolated trochleoplasty for recurrent patellar dislocation has lower outcome and higher residual instability compared with combined MPFL and trochleoplasty: a systematic review*	2019	Review	4	N/A	N/A	N/A
34 (23)	**Balcarek, P; Zimmermann, F** *Deepening trochleoplasty and medial patellofemoral ligament reconstruction normalise patellotrochlear congruence in severe trochlear dysplasia*	2019	Prospective cohort study	3	22	52	23
35 (20)	**Nelitz, M; Dreyhaupt, J; Williams, SRM** *No Growth Disturbance After Trochleoplasty for Recurrent Patellar Dislocation in Adolescents With Open Growth Plates*	2018	Case series	4	14	61	23
36 (18)	**Laidlaw, MS; Feeley, SM; Ruland, JR; Diduch, DR** *Sulcus‐Deepening Trochleoplasty and Medial Patellofemoral Ligament Reconstruction for Recurrent Patellar Instability*	2018	Surgical technique	5	N/A	N/A	N/A
37 (17)	**Neumann, MV; Stalder, M; Schuster, AJ** *Reconstructive surgery for patellofemoral joint incongruency*	2016	Case series	4	8	57	23
38 (16)	**Dejour, DH; Deroche, É** *Trochleoplasty: Indications in patellar dislocation with high‐grade dysplasia. Surgical technique*	2022	Surgical technique	5	N/A	N/A	N/A
39 (16)	**Carstensen, SE; Feeley, SM; Burrus, MT; Deasey, M; Rush, J; Diduch, DR** *Sulcus Deepening Trochleoplasty and Medial Patellofemoral Ligament Reconstruction for Patellofemoral Instability: A 2‐Year Study*	2020	Case series	4	9	56	24
40 (15)	**Leclerc, JT; Dartus, J; Labreuche, J; Martinot, P; Galmiche, R; Migaud, H; Pasquier, G; Putman, S** *Complications and outcomes of trochleoplasty for patellofemoral instability: A systematic review and meta‐analysis of 1000 trochleoplasties*	2021	Systematic review and meta‐analysis	4	N/A	N/A	N/A
41 (15)	**Biedert, RM** *Combined deepening trochleoplasty and supracondylar external rotation osteotomy for recurrent patellar instability in patients with trochlear dysplasia and increased femoral antetorsion*	2020	Case series	4	12	70	22
42 (15)	**Carstensen, SE; Menzer, HM; Diduch, DR** *Patellar Instability: When is Trochleoplasty Necessary?*	2017	Review	4	N/A	N/A	N/A
43 (15)	**Pesenti, S; Blondel, B; Armaganian, G; Parratte, S; Bollini, G; Launay, F; Jouve, JL** *The lateral wedge augmentation trochleoplasty in a pediatric population: a 5‐year follow‐up study*	2017	Case series	4	10	54	19
44 (15)	**Beaufils, P; Thaunat, M; Pujol, N; Scheffler, S; Rossi, R; Carmont, M** *Trochleoplasty in major trochlear dysplasia: current concepts*	2012	Review	4	N/A	N/A	N/A
45 (14)	**Falkowski, AL; Camathias, C; Jacobson, JA; Magerkurth, O** *Increased Magnetic Resonance Imaging Signal of the Lateral Patellar Facet Cartilage A Functional Marker for Patellar Instability?*	2017	Case series	4	12	45	26
46 (12)	**Vogel, LA; Pace, JL** *Trochleoplasty, Medial Patellofemoral Ligament Reconstruction, and Open Lateral Lengthening for Patellar Instability in the Setting of High‐Grade Trochlear Dysplasia*	2019	Surgical technique	5	N/A	N/A	N/A
47 (12)	**Zaki, SH; Rae, PJ** *Femoral trochleoplasty for recurrent patellar instability: a modified surgical technique and its medium‐term results*	2010	Case series	4	10	56	23
48 (11)	**von Engelhardt, LV; Weskamp, P; Lahner, M; Spahn, G; Jerosch, J** *Deepening trochleoplasty combined with balanced medial patellofemoral ligament reconstruction for an adequate graft tensioning*	2017	Case series	4	15	63	22
49 (10)	**Kaiser, D; Trummler, L; Götschi, T; Waibel, FWA; Snedeker, JG; Fucentese, SF** *Patellofemoral instability in trochleodysplastic knee joints and the quantitative influence of simulated trochleoplasty ‐ A finite element simulation*	2021	Biomechanical study	5	N/A	N/A	N/A
50 (10)	**Koch, PP; Fuchs, B; Meyer, DC; Fucentese, SF** *Closing wedge patellar osteotomy in combination with trochleoplasty*	2011	Case report	5	N/A	N/A	N/A

Abbreviations: LOE, level of evidence; MCMS, Modified Coleman Methodological Score; MINORS, Methodological Index for Non‑Randomised Studies; MQCSRE, Radiologic Methodology and Quality Scale.

**Figure 3:**
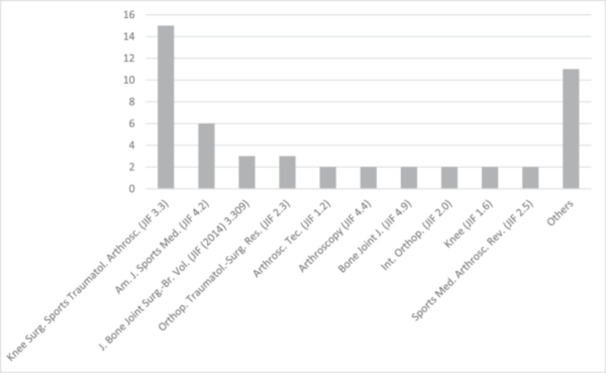
Distribution of the top 50 studies by the journals of publication and their Journal Impact Factor of 2021 (JIF). Knee Surgery, Sports Traumatology, Arthroscopy (Knee Surg Sports Traumatol Arthrosc), American Journal of Sports Medicine (Am J Sports Med), Journal of Bone and Joint Surgery, British Volume (J Bone Joint Surg Br), Orthopaedics & Traumatology: Surgery & Research (Orthop. Traumatol‐Surg. Res.), Arthroscopy Techniques (Arthrosc. Tec.), Arthroscopy (Arthroscopy), The Bone & Joint Journal (Bone Joint J), International Orthopaedics (Int. Orthop), Knee (Knee), Sports Medicine and Arthroscopy Review (Sports Med. Arthrosc. Rev.).

The top 50 most cited articles were published in 21 journals. In total, 15 articles (30%) were published in the journal ‘Knee Surgery Sports Traumatology Arthroscopy’ (KSSTA), followed by 6 articles (12%) published in the ‘American Journal of Sports Medicine’ (Figure 3). The Bone & Joint Journal is the highest‐ranked journal, considering its JIF of 4.9 and JCI of 2.15. KSSTA ranks fifth with a JIF of 3.3 and a JCI of 1.47, while AJSM is ranked fourth by JIF (4.2) and third by JCI (1.83). The mean JIF was 3.0 ± 1.2, with 58% of the studies published in a journal with a JIF of 3.3 or higher. The mean JCI was 1.30 ± 0.56.

**Figure 4:**
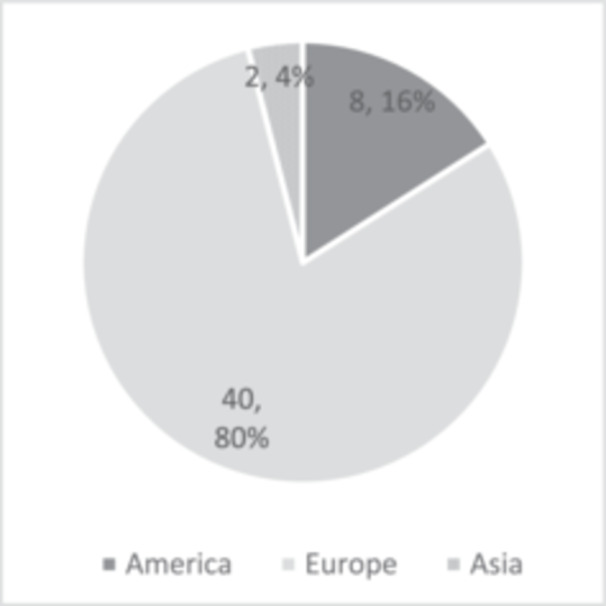
Continents and countries of main research conducting institute.

**Figure 5:**
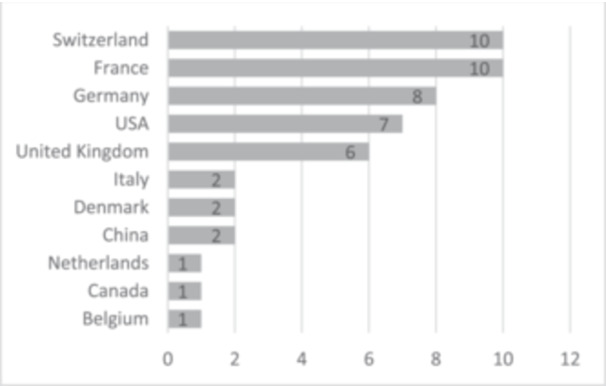
Continents and countries of main research conducting institute.

The majority of studies were published by European institutions (*n* = 40; 80%) (Figure 4), with France and Switzerland contributing 10 publications each, followed by Germany (*n* = 8) and the United Kingdom (*n* = 6) (Figure 5). Seven publications (14%) originated from institutions in the United States (US), and one (2%) was published by a Canadian institution. Notably, between 2000 and 2009, no studies from the US or Canada appeared in the top 50. However, between 2010 and 2019, 18.9% (*n* = 7) of the top studies were conducted in North America, and from 2020 to 2024, 20% (*n* = 1) were published by American institutions. This reflects a modest increase in North American contributions, though the change did not reach statistical significance (*p* > 0.05).

Case series (*n* = 25; 50%) and SRs and meta‐analyses (*n* = 16; 32%) represented the most prevalent study designs. The majority of articles were classified as LOE IV (*n* = 41; 82%). A comparison of LOE across the last three decades revealed no statistically significant differences (*p* > 0.05). Notably, there were no studies with a LOE higher than III, with only one study (*n* = 1; 2%) reaching this level. In 23 of the 50 studies (*n* = 46%), TP was the sole intervention. Several studies included TP with only one specific concomitant procedure, such as medial patellofemoral ligament (MPFL) reconstruction (*n* = 14, 28%), extensor apparatus balancing (*n* = 1), supracondylar external rotation osteotomy (*n* = 1) or closing wedge osteotomy of the patella (*n* = 1). All other studies incorporated TP alongside a heterogeneous mix of additional procedures (*n* = 10, 20%) within their patient cohorts.

**Figure 6:**
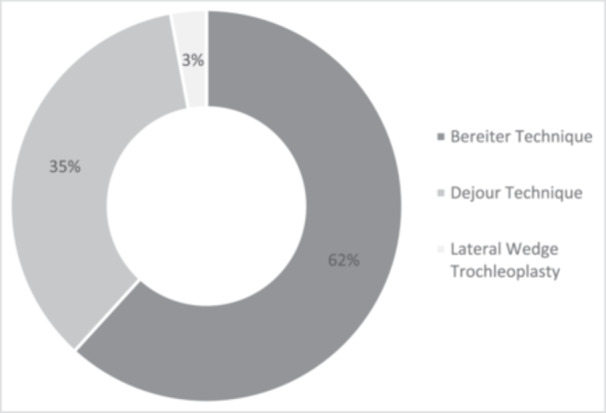
Distribution of the surgical technique described in articles.

Various techniques for performing TP have been described in the literature. SRs (*n* = 16) frequently did not specify the technique used in the included studies. Among the remaining studies, the Bereiter technique (*n* = 21; 61.8%) and the Dejour technique (*n* = 12; 35.3%), along with modifications of these approaches, were the most commonly represented in this review (Figure 6). Additionally, one study employed a lateral wedge TP. Notably, two studies reported the use of the Bereiter technique in an arthroscopic manner, highlighting the evolving nature of surgical approaches in this domain.

### Quality assessment

The methodological quality of 26 studies was assessed, while 24 studies—including 16 SRs and meta‐analyses, 4 articles on surgical techniques, 2 biomechanical studies, 1 histological examination and 1 case report—were excluded from evaluation, as the selected scoring systems were not designed for these study types (Table 2). The mean quality scores were 60.8 ± 9.8 for MCMS (*n* = 26), 11.1 ± 2.9 for MINORS (*n* = 26) and 22.5 ± 2.1 for MQCSRE (*n* = 25). Ten studies (43.5%) attained a MINORS score of 12 (or 18 for comparative studies) or higher, which is a commonly recognised threshold for high‐quality research. Interrater assessments showed fair agreement for MCMS (*κ* = 0.36) and moderate agreement for MINORS (*κ* = 0.54) and MQCSRE (*κ* = 0.47), indicating consistency between the two raters [10].

**Table 2:** Mean scores during the three past decades: Modified Coleman Methodology Score (MCMS) [4], Methodological Index for Non‐Randomised Studies (MINORS) [20] and Radiologic Methodology and Quality Scale (MQCSRE) [2].

Decade of publication	2000–2009	2010–2019	2020–2024
MCMS	59.33 ± 10.67	61.00 ± 10.06	63.00 ± 9.90
MINORS	9.17 ± 1.60	11.83 ± 3.05	10.50 ± 2.12
MQCSRE	21.40 ± 3.64	22.78 ± 1.59	23.00 ± 1.41

All mean scores, with the exception of MINORS scores from 2010 to 2019 compared to 2020 to 2024, showed an upward trend over the past three decades, as detailed in Table 2. This finding suggests an enhancement in methodological quality, particularly in studies assessed by the MINORS criteria during this time frame.

The comparison of the three quality scores between articles published by institutions based in America, Europe and Asia revealed no statistically significant differences (*p* > 0.05). However, Germany demonstrated a significantly higher mean MINORS score of 13.83 ± 4.67, compared to the mean score of 10.30 ± 1.49 from the rest of the countries (*p* = 0.028). In contrast, the other two scores, MCMS and MQCSRE, showed no statistically significant differences between countries (*p* > 0.05).

### Citation counts

A substantial proportion of the citations (1551; 62.5%) originated from publications in three prominent journals. Of these, 803 citations (32.4%) were derived from articles published in *Knee Surgery, Sports Traumatology, Arthroscopy*, 444 citations (17.9%) came from *The Journal of Bone and Joint Surgery* (combining both the American and British volumes), and 304 citations (12.3%) were attributed to articles from *The American Journal of Sports Medicine*. An examination of the past three decades reveals that 37 out of 50 records (74%) were published between 2010 and 2019, corresponding to a concentration of 1561 citations (62.9%) during this decade. The average number of citations per article from this period reached 42.19 ± 33.20. In contrast, articles published between 2000 and 2009 received a significantly higher average citation count of 106.00 ± 39.45 per article, while those from 2020 to 2024 had a mean citation count of 14.40 ± 2.51 per article. These differences were statistically significant (*p* < 0.001).

Only marginal variations were observed when analysing citation density across the decades. The mean citation density was 6.01 ± 2.07 for articles from 2000 to 2009, compared to 4.90 ± 2.77 for the 2010–2019 period, and 4.81 ± 1.88 for articles from 2020 to 2024. These differences, however, did not reach statistical significance (*p* > 0.05). The study with the highest citation density, at 13.35 citations per year, was also the most cited overall (187 citations), authored by Dejour and Saggin. This indicates that while citation counts vary significantly by decade, the rate of citations accumulated per year remained relatively stable across time [6].

Interestingly, no significant correlation was found between citation count and the three study quality scores (MCMS: *p* = 0.543; MINORS: *p* = 0.122; MQCSRE: *p* = 0.941) or LOE (*p* > 0.05). Furthermore, citation counts and citation densities showed no significant variation based on the country of origin (*p* > 0.05). Similarly, when comparing continents (America, Europe and Asia), there were no significant differences in mean citation counts or citation density per article (*p* > 0.05).

A higher mean citation count was observed for studies employing the TP technique described by David Dejour compared to the thin flap technique described by Bereiter (69.8 ± 54.6 vs. 50.43 ± 44.0; *p* = 0.178). Dejour's TP was also cited more often per year (6.16 ± 2.97 vs. 4.42 ± 2.43; *p* = 0.082). However, these differences were not statistically significant.

## DISCUSSION

The primary objective of the current analysis of the 50 most cited studies on TP was to assess the methodological quality of publications in this field and to determine whether the quality of these studies correlated with their citation frequency. No significant correlation between citation count and methodological study quality scores (MCMS, MINORS and MQCSRE) or LOE was discovered.

The scarcity of Levels I, II and III studies was notable, with Level IV studies comprising 82% (*n* = 41) of analysed studies. No randomised controlled trials were identified, and only one prospective study (2%) was included. Additionally, a significant portion of the studies were SRs and meta‐analyses (*n* = 16; 32%), underscoring the need for research with higher methodological quality and levels of evidence in this domain. However, should be acknowledged that this is particularly challenging in surgical fields due to strict regulatory requirements and the inherent variability in indications and concomitant procedures, which limit the feasibility of conducting randomised and controlled trials [14]. Although no significant correlation was found between citation count or JIF and the methodological quality of the studies, a substantial portion of the top 50 studies (*n* = 37, 74%) were published in journals ranked in the top quartile of the orthopaedic subcategory based on impact factor. This highlights the reliance on journal reputation and author prestige as proxies for research quality by orthopaedic surgeons. It underscores the need for an objective tool to assess methodological quality, as the journal may not always reflect the true rigour or scientific merit of the research [5]. Therefore, it is advisable to carefully consider the study methodologies when selecting papers for citation. The LOE within the top 50 was achieved by the study published by Balcarek and Zimmermann [3], conducting a comparative analysis of preoperative and postoperative patellofemoral morphology using MRI in patients who underwent TP and MPFL reconstruction, matched with control patients of similar age and gender. This exemplary study highlights the importance of robust methodologies in providing reliable, high‐quality scientific evidence for clinical decision‐making. It is noteworthy that the only study with this degree of scientific evidence compares radiographic parameters and not clinical outcomes.

Additionally, it was hypothesised that factors such as the country of origin, year of publication, journal of publication, and surgical techniques might correlate with citation count, citation density, and methodological quality scores in the top 50 most cited studies. A significant difference was observed only in the mean MINORS score, with papers from German institutes achieving a higher average compared to the overall mean MINORS score of studies from other countries (13.83 ± 4.67 vs. 10.30 ± 1.49; *p* = 0.028). Additionally, while methodological quality scores generally improved over the past three decades, the only statistically significant increase was seen in the MINORS score for studies published between 2010 and 2019, compared to the preceding decade (11.83 ± 3.05 vs. 9.17 ± 1.60; *p* = 0.18). This highlights a modest yet notable enhancement in the methodological rigour of studies over time.

Historically, TP has been predominantly a European‐centred topic, with the majority of studies (*n* = 40; 80%) originating from European institutions, particularly in France, Switzerland, the United Kingdom and Germany. Comparisons across decades suggest a growing interest from American institutions. Additionally, there has been an observable trend of increasing publications per 5‐year period, signalling a growing interest and expanding research focus on TP in recent years.

Studies utilising the TP technique first described by Dejour demonstrated a higher mean citation count (69.8 ± 54.6) compared to those using Bereiter's thin flap technique (50.43 ± 44.0); these differences were not statistically significant. Interestingly, despite the higher citation count for Dejour's technique, Bereiter's method was used in 21 of the top 50 studies, whereas Dejour's technique appeared in only 12, suggesting a broader scientific interest in Bereiter's approach. Leclerc et al. demonstrated in an SR and meta‐analysis, that Dejour's deepening TP is the most effective, with only 1 recurrence in 349 knees (0.28%) versus 18 reported recurrences (*n* = 18/552; 3.2%) when using Bereiter's technique [11].

Dejour and Saggin published the most cited study in our report [6], accumulating 187 citations and achieving the highest citation density, with 13.4 citations per year. The study's significant citation count likely stems from its comprehensive description of Dejour's original surgical technique for sulcus‐deepening TP. Additionally, the authors conducted an extensive review of radiologic features, surgical indications, complications, and outcomes from previous studies on TP, contributing to its influence in the field. Due to the nature of the study, it was not suitable for the selected quality scoring systems, and thus, no methodological score was recorded.

von Knoch et al. [23] ranked second with 169 citations and a yearly citation density of 9.4 with their 4‐ to 14‐year follow‐up on TP. The mean follow‐up period was comparatively long, with 8.3 years, resulting in middle of the field MINORS and MQCSRE scores of 61 and 23. Despite the study's influence, the MCMS score was notably low, scoring only 8 points and thus ranking last among the evaluated studies.

Ranking third, with 134 citations and a density of 7.1 citations per year, was the study by Schöttle et al. titled ‘Trochleaplasty for patellar instability due to TD: A minimum 2‐year clinical and radiological follow‐up of 19 knees’ [19], being in the middle to lower third in quality scores (MCMS = 10, MINORS = 60 and MQCSRE = 22). In this study, radiological features using CT scans and clinical parameters were evaluated in 16 patients at a mean follow‐up of 3 years. A low number of cases and a short follow‐up period were the main factors contributing to the lower quality scores of this study.

Overall, low MCMS was influenced by small sample sizes (only two studies with more than 80 patients [12, 13]), short mean follow‐up periods (two studies with a mean follow‐up longer than 6 years [15, 17]) and weak study designs, as only one study [3] had a LOE of III. Lower MINORS scores resulted from non‐prospective data collection and the absence of control groups, which significantly impacted the overall MINORS score. MQCSRE scores were relatively consistent; however, the lack of accountability for test‐review bias and inter‐ and intrarater reliability often contributed to lower scores.

This study has several limitations. Given the substantial number of SR, biomechanical or anatomical studies and descriptions of surgical techniques included in the final selection, only 26 studies were subjected to methodological quality assessment. Comparisons among these studies may also be confounded by variations in surgical techniques and concomitant procedures, such as MPFL reconstruction, osteotomies, or extensor apparatus balancing. Moreover, this analysis exclusively utilised search results from WoS and Scopus. A further limitation stems from the inherent subjectivity of certain items on the methodological grading scales. The MCMS score was designed to compare the methodological quality of cohort studies and tends to favour prospective cohort studies. Most of the studies in this top 50 list are case–control or retrospective cohort studies and, as a result, they may be graded up to 15 points lower due to their study type. The MINORS score favours studies with a long follow‐up period and a detailed rehabilitation protocol. Terms such as ‘well‐described’ rehabilitation protocol versus ‘described without complete detail’ within the MCMS scoring rubric introduced subjective variability. Similar problems were observed with the MINORS scale, where criteria like ‘a clearly stated aim’ or ‘follow‐up period appropriate to the aim of the study’ were evaluated to rate study quality, potentially resulting in divergent interpretations of a publication's quality by different investigators. To mitigate this bias, two investigators independently assessed all articles using both scales and reached a consensus in cases of scoring discrepancies. Furthermore, scientific writing itself inherits certain effects, which could bias the results of a study examining citation counts. Researchers often tend to cite highly cited articles more frequently in their manuscripts, creating a snowball effect that can distort the results [9]. This effect is difficult to quantify or estimate.

## CONCLUSION

High citation counts did not correlate with superior methodological quality. Despite the lack of studies with high levels of evidence and robust methodological strength, there is an increasing scientific interest in TP as a treatment for patients suffering from PFI. This review of the top 50 most cited studies on TP serves as a valuable resource for orthopaedic surgeons, offering a foundational tool to evaluate the most significant and impactful academic contributions in this evolving field.

## AUTHOR CONTRIBUTIONS

Conception: Armin Runer, Alexander Pfarrmaier and Romed P. Vieider. Data curation: Alexander Pfarrmaier and Rodrigo Sanchez. Data analysis: Alexander Pfarrmaier. Preparing the draft of the manuscript: Alexander Pfarrmaier, Rodrigo Sanchez and Armin Runer. Review and Editing: Alexander Pfarrmaier, Rodrigo Sanchez, Romed P. Vieider, Armin Runer, Lukas N. Muench, Lukas Willinger and Sebastian Siebenlist. Supervision: Armin Runer. All authors read and approved the final manuscript.

## CONFLICT OF INTEREST STATEMENT

The authors declare no conflicts of interest.

## ETHICS STATEMENT

No ethical approval was required for this literature review.

## Data Availability

The data sets used and/or analysed during the current study are available from the corresponding author on request.
